# Toenail and serum levels as biomarkers of iron status in pre- and postmenopausal women: correlations and stability over eight-year follow-up

**DOI:** 10.1038/s41598-023-50506-5

**Published:** 2024-01-19

**Authors:** Ann Von Holle, Katie M. O’Brien, Dale P. Sandler, Robert Janicek, Margaret R. Karagas, Alexandra J. White, Nicole M. Niehoff, Keith E. Levine, Brian P. Jackson, Clarice R. Weinberg

**Affiliations:** 1 Biostatistics and Computational Biology Branch National Institute of Environmental Health Sciences, Mail Drop A3-03, P.O. Box 12233, Research Triangle Park, Durham, NC 27709 USA; 2 Epidemiology Branch, National Institute of Environmental Health Sciences, Research Triangle Park, NC USA; 3 Advanced Research and Diagnostic Laboratory, University of Minnesota, Minneapolis, MN USA; 4 Department of Epidemiology, Geisel School of Medicine at Dartmouth, Dartmouth College, Hanover, NH USA; 5 RTI International, Research Triangle Park, NC USA; 6 Department of Earth Sciences, Dartmouth College, Hanover, NH USA; 7 Present Address: Ontada, Durham, NC USA

**Keywords:** Biomarkers, Biomarkers, Outcomes research, Epidemiology

## Abstract

Iron status is often assessed in epidemiologic studies, and toenails offer a convenient alternative to serum because of ease of collection, transport, and storage, and the potential to reflect a longer exposure window. Very few studies have examined the correlation between serum and toenail levels for trace metals. Our aim was to compare iron measures using serum and toenails on both a cross-sectional and longitudinal basis. Using a subset of the US-wide prospective Sister Study cohort, we compared toenail iron measures to serum concentrations for iron, ferritin and percent transferrin saturation. Among 146 women who donated both blood and toenails at baseline, a subsample (59%, n = 86) provided specimens about 8 years later. Cross-sectional analyses included nonparametric Spearman’s rank correlations between toenail and serum biomarker levels. We assessed within-woman maintenance of rank across time for the toenail and serum measures and fit mixed effects models to measure change across time in relation to change in menopause status. Spearman correlations at baseline (follow-up) were 0.08 (0.09) for serum iron, 0.08 (0.07) for transferrin saturation, and − 0.09 (− 0.17) for ferritin. The within-woman Spearman correlation for toenail iron between the two time points was higher (0.47, 95% CI 0.30, 0.64) than for serum iron (0.30, 95% CI 0.09, 0.51) and transferrin saturation (0.34, 95% CI 0.15, 0.54), but lower than that for ferritin (0.58, 95% CI 0.43, 0.73). Serum ferritin increased over time while nail iron decreased over time for women who experienced menopause during the 8-years interval. Based on cross-sectional and repeated assessments, our evidence does not support an association between serum biomarkers and toenail iron levels. Toenail iron concentrations did appear to be moderately stable over time but cannot be taken as a proxy for serum iron biomarkers and they may reflect physiologically distinct fates for iron.

## Introduction

Iron is a metal essential to human life—but toxic at high levels. Body iron levels and stores are a focus of health research given their associations with many health outcomes such as iron deficiency^1^, iron overload from hemochromatosis^2^, cancer^3–6^, liver disease^7^, diabetes^8,9^, and adverse cardiac outcomes such as cardiomyopathy^10^. Serum ferritin, iron, and transferrin saturation are among the most common serum iron outcomes used to measure circulating and stored iron in the human body and to assess iron status.^11^ Each of these levels indicates a different aspect of iron metabolism: serum iron is the amount of iron circulating in the blood; ferritin is an iron protein that binds and sequesters intracellular iron and is used as an indicator of iron storage^6,12,13^; percent transferrin saturation represents the percent of transferrin (plasma iron transport protein)^14^ in the blood that is bound to iron. These levels require blood draw(s), which can serve as a disincentive for study participants.

Given the resources required to collect, freeze, and store the blood as well as potential nonparticipation of people not amenable to blood draws, collection of serum samples may not be practicable for larger-scale studies. Also, there are analytic issues associated with blood serum samples, including that the blood levels are subject to homeostasis mechanisms of regulation^15,16^, day-to-day variability^17–20^, contamination from sampling needles and tubes^21,22^, and hemolysis during blood collection^23,24^. Toenails offer an alternative that is both less invasive to collect and easier to store, with no requirement to freeze samples^3,25^, Toenails also allow the evaluation of longer term exposures^26^ and may be less susceptible to contamination than hair and fingernails^27^. Although, like serum samples, nail samples also have analytic issues associated with them, such as a minimum weight for analyses, usually 10–25 mg^28^, and nail sample preparation including washing to remove contaminants prior to analyses^28,29^.

Determining the degree to which toenail iron levels are associated with certain types of serum iron levels, such as ferritin and transferrin saturation, is critical if investigators want to treat toenail levels as a proxy for biomarkers based on serum. While there is evidence supporting similarities between levels in serum and toenails for metals such as selenium^30^, there is little evidence comparing iron levels in serum and toenails^26^ and some studies provide descriptive data on levels in toenails and serum^31,32^. Similarly, evidence is lacking for other elements that are known to bioconcentrate in nails, such as mercury and arsenic. Furthermore, comparisons of repeated levels are also sparse^33^, and estimates of change and stability can provide evidence of the similarity or lack thereof between the two matrices for iron levels. Our aim was to compare iron levels in toenails with three serum biomarkers: iron, ferritin and percent transferrin saturation, on both a cross-sectional and longitudinal basis.

## Methods

### Study population

**Figure 1:**
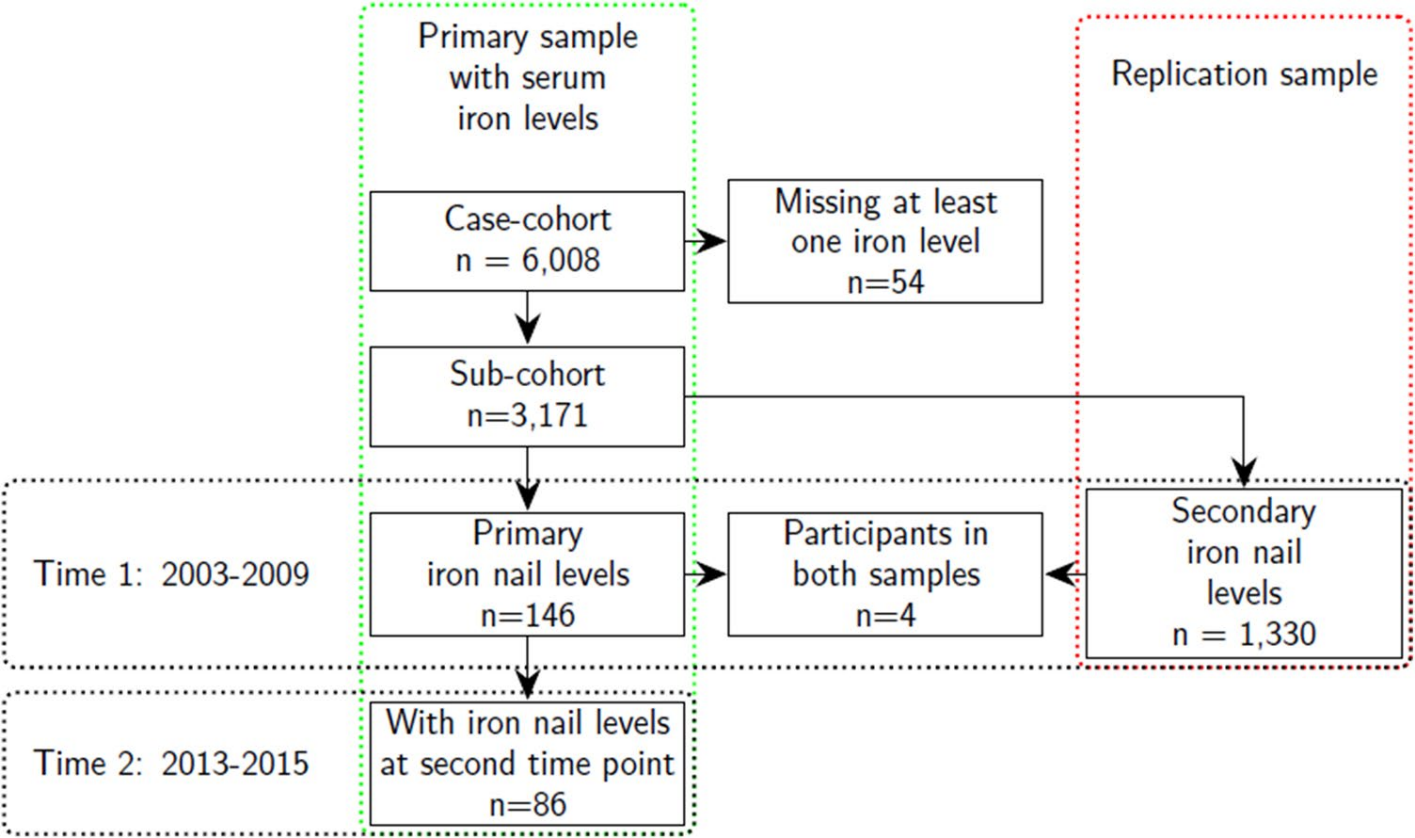
Sample flow diagram. The green lines indicate the flow diagram for the primary sample with participants having both serum and toenail iron levels. The red lines enclose th replication sample with baseline values for participants with both serum and toenail iron levels.

We used subsamples from the US-wide prospective Sister Study cohort^34^ of women who, at enrollment, were between ages 35–74 years. The current analysis is from data release 8.1 (December, 2020) of the Sister Study,^34^ a prospective cohort of 50,884 women who had not been diagnosed with breast cancer prior to enrollment (2003–2009) but had at least one sister who had been diagnosed with breast cancer. We used a subcohort (n = 3171) sampled for a case-cohort study of iron and breast cancer^35^ with at least one serum iron value (Fig. 1). Our primary analyses included the subsample of participants (n = 146) who had data on all three serum levels and toenail iron levels at baseline from an ancillary study of young-onset cases^36,37^. A subsample (59%, n = 86) of those participants provided both specimens again about 8 (interquartile range (IQR): 7,9) years later. This cohort also includes a group of women who were premenopausal at baseline and postmenopausal at the follow-up, and their repeated levels can provide insight into how each matrix captures the accompanying iron store change occurring with this transition^38^.

We used a second sample from another subset (n = 1330)^39^, which included participants with at least one of the three serum levels and toenails collected only at baseline (2003–2009) to replicate our baseline cross-sectional findings. Using levels that were batch-corrected with mixed effects models^37^, we compared their correlation levels at baseline with those from the primary sample. Four of the participants in this sample had also been included in the subcohort sample used in the primary analysis.

### Laboratory sample analysis

Toenail clippings were self-collected by the study participants, who were instructed to obtain clippings from each toenail after removing nail polish^36^. Analysis of the toenail clippings has been described elsewhere for both the primary and replication sample^36,39^. For the primary sample clippings, they were washed with acetone, Triton X-100 surfactant, and deionized water, then left to dry in a HEPA filtered dry box for a minimum 72 h or until the nails were completely dry. After washing the nail clippings, they were air dried and digested in an acid solution of 9:1 HNO_3_:HCl^36^. Following this process, and if the sample exceeded a minimum nail mass of 10 mg, the samples were diluted in deionized water and analyzed using inductively coupled plasma mass spectrometry (Agilent 8800 ICP-MS/MS; Santa Clara, CA) in collision (helium) mode. A sample of digest blanks had a median Fe of 0.7 ppb.

The secondary replication sample followed similar analytic steps as the primary sample. The toenail clippings were washed with Triton X-100 surfactant, HPLC grade acetone, and deionized water^39^. After the washing process, the nails were digested in both a nitric and hydrochloric acid solution and hydrogen peroxide, and analyzed using and inductively coupled plasma mass spectrometry (Thermo Scientific iCAP™ RQ ICP-MS, Waltham, MA)^39^. Batch-level quality control included laboratory reagent calibration blanks for samples above a limit of detection for the lowest calibration standard (500 ng/g). The coefficient of variation in a subsample of iron in the reference material was 18% (n = 72).

A Roche Cobas 6000 Chemistry analyzer (Roche Diagnostics, Mannheim, Germany) was used to perform all serum analyses^35^ with Roche reagents and calibrators according to manufacturer standards. Iron and unsaturated iron binding capacity (UIBC) were measured with colorimetric assays and ferritin was measured with a particle enhanced immunoturbidimetric assay. Samples were tested for hemolysis, icterus and lipemia using a spectrophotometric method and were not reported if they did not meet the manufacturer standards. Quality control occurred on a daily basis at the beginning and end of testing for the serum samples and included a pooled laboratory serum sample and a Roche product. Sample quality control also included calculations of interassay coefficients of variation, which were 2.5% for serum iron, 2.8% for serum ferritin, and 3.3% for UIBC^35^.

All participants provided written informed consent. The institutional review board of the National Institutes of Health, Bethesda, Maryland (United States of America) provides study approval and oversight (protocol number 02EN271). All methods for this study were carried out in accordance with relevant guidelines and regulations.

### Statistical methods

Descriptive statistics included median and interquartile ranges for continuous variables and frequencies with percentages for the categorical variables.

### Cross-sectional analyses

To compare iron levels in toenails with three serum iron levels (iron, ferritin and percent transferrin saturation), we used both non-parametric and parametric methods. For cross-sectional comparisons, we visually examined scatter plots of toenail versus serum observations to assess the association between toenails and serum levels. We used non-parametric Spearman rank correlation coefficients to assess the cross-sectional associations between the paired observations, both at the baseline and at follow-up. The coefficient of variation (CV), the ratio of the standard deviation among the samples and the mean, served as a measure of dispersion for toenails and serum. However, differences between the CV based on serum levels versus toenail levels must be interpreted carefully, as the CV reflects both person-to-person differences and variation due to measurement errors.

### Longitudinal analyses

We stratified the longitudinal analyses by menopause status at baseline/follow-up to include three comprehensive groups: premenopausal/premenopausal, premenopausal/postmenopausal, and postmenopausal/postmenopausal. To compare the two repeated levels, we first calculated the paired rank-based Spearman correlations and intraclass correlation coefficients between baseline and follow-up separately for the toenail and serum levels. Next, we subtracted the natural log-transformed baseline measure from the natural log-transformed follow-up measure for each of the serum and toenail levels. We then calculated a correlation coefficient between each of the serum differences and the corresponding toenail differences, to estimate the correspondence between changes over time for each serum-based measure versus toenails. A coefficient close to one would indicate a similarity between the serum and toenail changes from baseline to follow-up times. An additional analysis included calculating these correlation coefficients for the pairs of serum levels.

Follow-up times varied, and there may be changes in iron status over age-time that can also depend on menopausal status. To accommodate different age-time intervals, we also fit a mixed effects regression model with natural log-transformed iron levels as the outcome and covariates including an intercept, age, separate random effects for the participant’s blood measures and toenail measure, an indicator variable for toenails (versus serum levels), and a product term between that indicator and age (Supplemental text). The log-transform of all outcome variables enables an interpretation of 100 times one less than the exponentiated age regression coefficient as an approximate percent change in the outcome per one year change in age^40,41^. The mixed effects model also included correlations between the random effects, autocorrelated errors for time, and a random intercept. Iron stores can depend on menopause status^42–44^, i.e. whether monthly bleeding has ceased, and we consequently carried out separate analyses for each of the three combinations of pre- and postmenopausal status at baseline and follow-up. In these analyses, menopause status was based on cessation of bleeding. For example, for women who reported premenopausal hysterectomy, the age at the hysterectomy was the point at which we considered the woman as postmenopausal in these models.

### Seasonal patterns in toenail levels

Following analyses of the observed toenail and serum iron levels, we also examined the Spearman correlation coefficients after removing seasonal changes in toenail iron levels. To assess and remove seasonal variation in toenail iron, we followed two steps. First, we fit toenail iron levels as an outcome in a simple linear regression with both sine and cosine of month times 2π/12 included as covariates^45^. In a second step, we used the residuals from that model to represent the seasonally-adjusted toenail iron levels, and calculated the cross-sectional and longitudinal Spearman correlations as defined above.

All analyses were done with R software^46^, version 4.0.1.

## Results

Women who had nail and serum values measured at baseline tended to be younger, with lower body mass index, had a higher level of education, and more likely to self-identify as Non-Hispanic White compared to the subcohort of women in the Sister Study sample (Table 1). In terms of baseline descriptive statistics, the subcohort from which this sample was derived was randomly sampled and consequently similar to the entire Sister Study sample^34^.

**Table 1:** Sample characteristics of baseline and follow-up.

Variable	Total	Serum and washed toenail iron levels	
		Baseline	Baseline and Follow-up
n	3171	146	86
Serum, baseline			
Iron, baseline (mcg/dL) (median [IQR])	93.00 [74.00, 115.00]	89.50 [71.00, 119.75]	86.50 [71.00, 119.75]
Ferritin, baseline (mcg/dL) (median [IQR])	67.00 [36.00, 112.00]	40.50 [24.00, 75.00]	38.00 [24.00, 74.00]
UIBC, baseline (median [IQR])	231.00 [197.00, 269.00]	241.00 [208.50, 291.50]	241.00 [206.50, 286.75]
TIBC, baseline (median [IQR])	328.00 [300.00, 360.00]	337.00 [306.25, 376.50]	337.00 [307.25, 380.75]
Transferrin saturation, baseline (%) (median [IQR])	29.00 [22.00, 36.00]	26.00 [21.00, 35.75]	26.00 [21.00, 35.00]
Serum, time 2			
Iron, time 2 (mcg/dL) (median [IQR])			93.00 [72.50, 111.00]
Ferritin, time 2 (mcg/dL) (median [IQR])			52.00 [28.00, 84.75]
UIBC, time 2 (median [IQR])			236.00 [210.00, 276.75]
TIBC, time 2 (median [IQR])			333.00 [302.75, 367.75]
Transferrin saturation, time 2 (%) (median [IQR])			27.00 [21.00, 34.75]
Washed toenails			
Iron, baseline (mcg/g) (median [IQR])	12.00 [8.55, 21.17]	11.78 [8.53, 20.85]	13.38 [8.39, 22.52]
Iron, time 2 (mcg/g) (median [IQR])	12.40 [7.59, 21.47]	12.51 [7.64, 21.44]	12.51 [7.59, 21.52]
Continuous			
Baseline age (years) (median [IQR])	55.60 [48.90, 62.00]	44.80 [41.90, 48.48]	44.00 [41.42, 46.77]
BMI (kg/m^2^) (median [IQR])	26.52 [23.22, 31.13]	25.67 [22.33, 31.58]	25.67 [21.95, 32.25]
Age difference, serum draws (years) (median [IQR])	7.60 [6.40, 8.70]	7.70 [6.70, 8.80]	7.70 [6.70, 8.88]
Age difference, toenail collection (years) (median [IQR])	7.70 [6.70, 8.90]	7.90 [6.70, 9.00]	7.90 [6.70, 8.95]
Postmenopausal at baseline = 1) Yes (%)			14 (16.3)
Education (%)			
HS degree or less	517 (16.3)	13 (8.9)	8 (9.3)
Associate or technical degree	450 (14.2)	22 (15.1)	14 (16.3)
Some college but no degree	612 (19.3)	19 (13.0)	7 (8.1)
Bachelor’s degree	836 (26.4)	53 (36.3)	36 (41.9)
Doctoral or Master’s degree	756 (23.8)	39 (26.7)	21 (24.4)
Race/ethnicity (%)			
Non-Hispanic White	2654 (83.7)	132 (90.4)	73 (84.9)
Non-Hispanic Black	278 (8.8)	9 (6.2)	9 (10.5)
Hispanic	152 (4.8)	3 (2.1)	3 (3.5)
Other	87 (2.7)	2 (1.4)	1 (1.2)

*IQR* Interquartile range.

### Cross-sectional analyses

**Figure 2:**
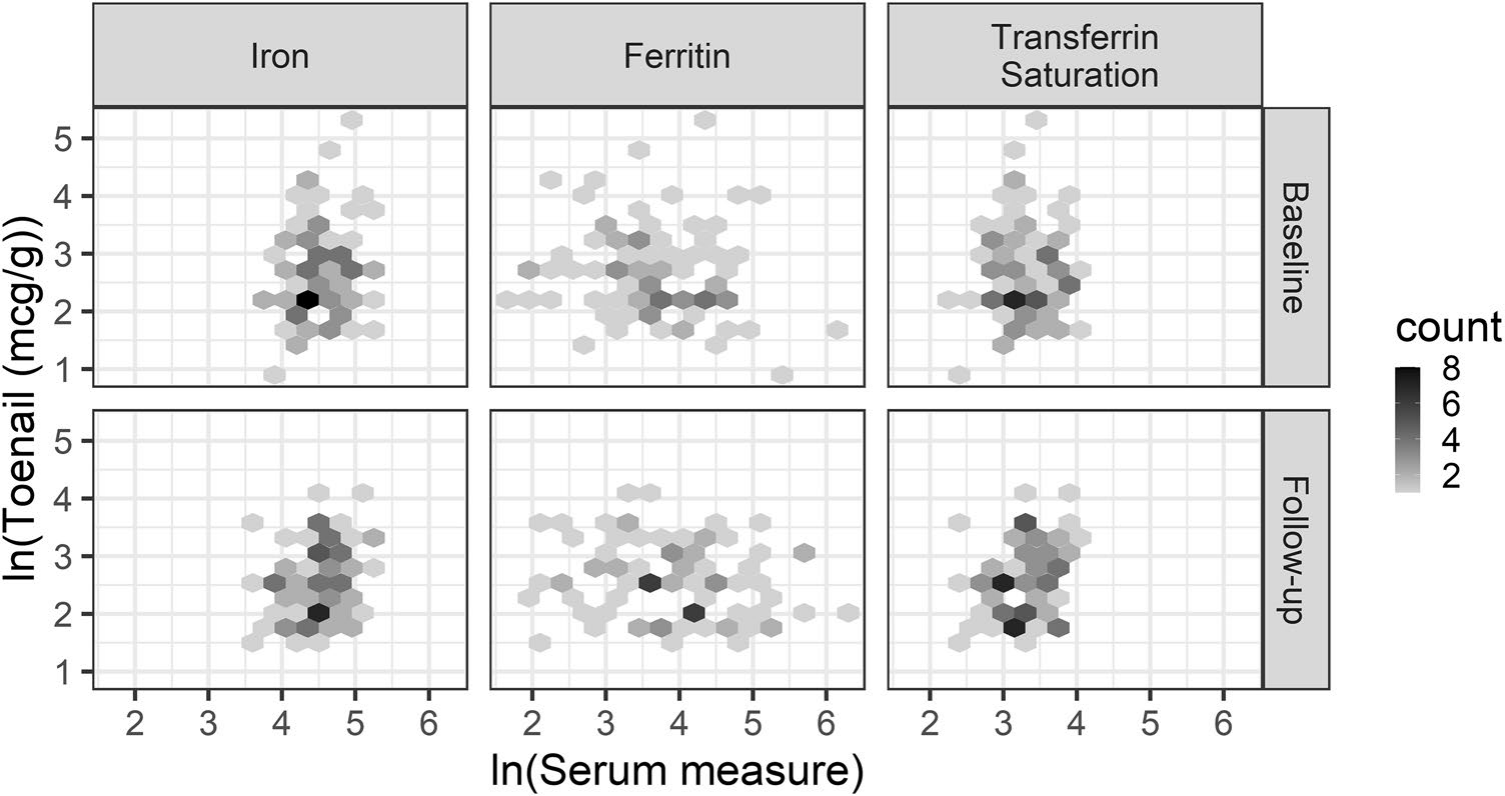
Scatter plots of natural log transformed washed toenail versus serum iron levels by time of collection.

Overall, toenail and serum iron values were not correlated. Spearman correlations at baseline (follow-up) between toenail and serum levels were 0.08 (0.09) for serum iron, 0.08 (0.07) for transferrin saturation, and − 0.09 (− 0.17) for ferritin (Table 2). These are rank-based correlations and natural log transformations produce the same estimates. Visual inspection of scatter plots at both baseline and follow-up (Fig. 2, Figure S1) further support the findings of low correlations.

**Table 2:** Spearmean correlations between nails and serum iron values^*a*^ and coefficient of variation for natural log-transformed levels at baseline and follow-up.

Serum measure	Coefficient of variation		Correlation coefficient between serum iron and nail measure	
	Baseline (n = 146)	Follow-up (n = 86)	Baseline (n = 146)	Follow-up (n = 86)
Toenail iron	0.27	0.24		
Serum iron	0.08	0.08	0.08	0.09
Ferritin	0.24	0.24	− 0.09	− 0.17
Transferrin saturation	0.12	0.11	0.08	0.07

Following natural log-transformation of values, the coefficient of variation (CV) for toenail levels at baseline (follow-up) (0.27 (0.24)) were similar to those for ferritin (0.24 (0.24)) and higher than those for serum iron (0.08 (0.08)) and transferrin saturation (0.12 (0.11)) (Table 2). The patterns for coefficients of variation, with higher CV for toenail and ferritin levels compared to the serum iron and transferrin saturation, were similar for the corresponding values in the replication sample (Table S1). Descriptive boxplots of natural log-transformed serum and nail iron levels in the primary and replication samples (Figs. S2, S3) were similar with a larger interquartile range for serum ferritin and nail iron than for serum iron and transferrin saturation.

### Longitudinal analyses

The Spearman correlation coefficient (95% confidence intervals) between the baseline and follow-up toenail iron levels (0.49, (0.31, 0.67)) was similar to that for ferritin (0.58 (0.44, 0.72)) and higher than those for serum iron (0.28, (0.06, 0.49)) and transferrin saturation (0.34 (0.13, 0.56)) (Table 3). All the intraclass correlation coefficients were less than 0.4. To assess correlations between changes in iron over time, we also estimated the correlation coefficient between toenail and serum iron levels for differences of natural log-transformed values at baseline and follow-up, and none of the three change correlations exceeded an absolute value of 0.07 (Fig. S4), indicating low correlation between changes in the levels over time. In contrast, the same change correlation coefficients between the pairs of serum levels were all greater than those with nail iron: 0.95 for serum iron/transferrin saturation, 0.18 for serum iron/ferritin, and 0.29 for serum ferritin/transferrin saturation.

**Table 3:** Spearman correlation coefficients (95% CI) between baseline and follow-up levels by nail and serum status.

Serum measure	Spearman correlation coefficient between two time points (n = 86)	Difference between nail iron and serum correlation coefficients (n = 86)	Intraclass correlation coefficients (ICC)
Serum iron	0.3 (0.09, 0.51)	0.17 (− 0.11, 0.45)	0.35
Ferritin	0.58 (0.43, 0.73)	− 0.11 (− 0.34, 0.12)	0.29
Transferrin saturation	0.34 (0.15, 0.54)	0.13 (− 0.13, 0.39)	0.20
Nail iron	0.47 (0.3, 0.64)		0.35

**Figure 3:**
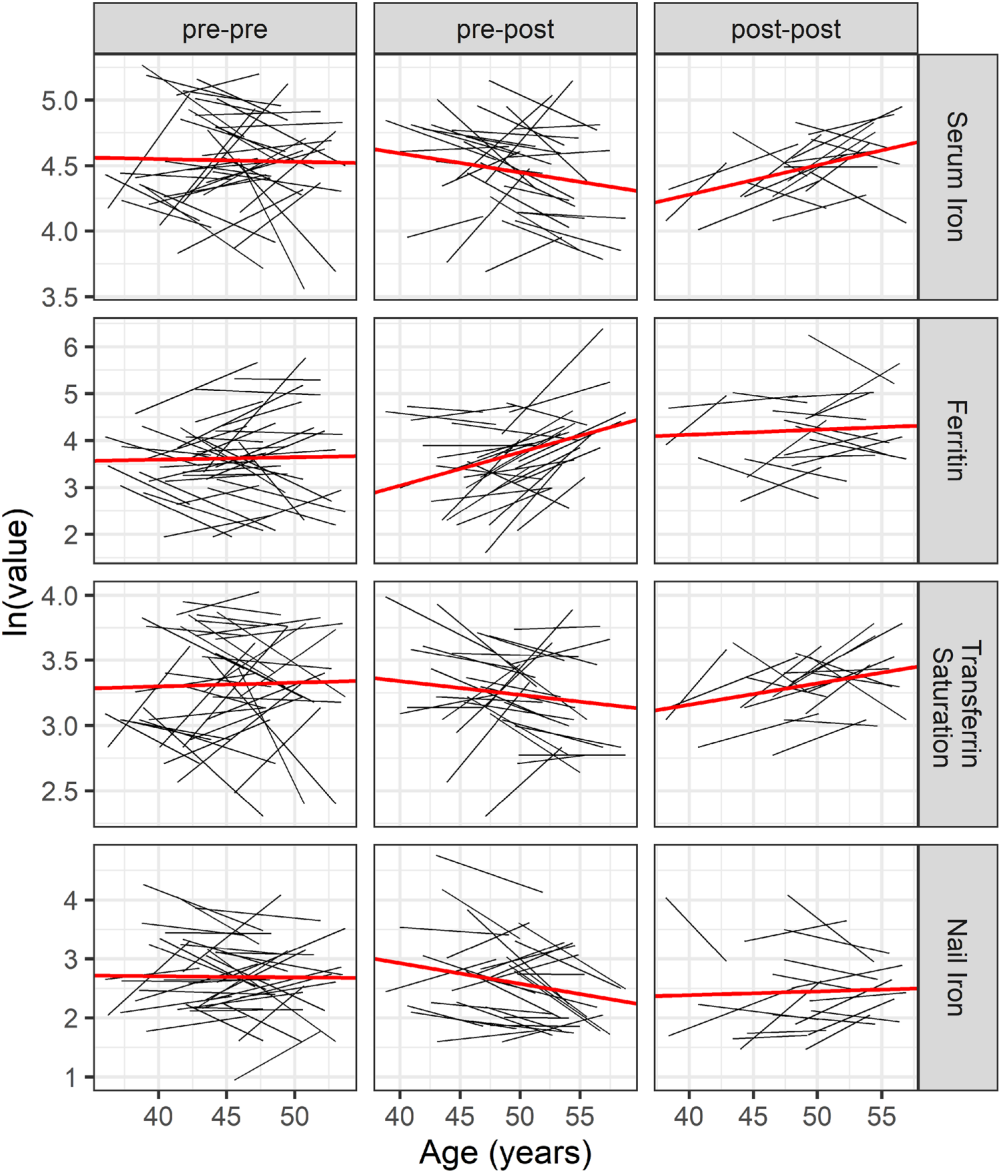
Nail and serum values (natural log transformed) over age-time by type of iron measure, menopause status combinations at baseline/follow-up, and case status. The solid red line indicates the fitted line from the mixed effects regression model, and each solid black line represents an individual.

The mixed effects model stratified by the three menopause status combinations at baseline versus follow-up (Table 4, Fig. 3), indicated that the group of women who were premenopausal at baseline and postmenopausal at follow-up had a 7.1% (95% CI: 3.7, 10.5) increase in ferritin per one year increase in age and a 1.4% (95% CI: − 2.9, 0.01) decrease in serum iron per year. In contrast, toenail iron for this group decreased an estimated 3.5% per year: (95% CI : − 6.1, − 0.8). Relative to the serum ferritin change for the pre- to postmenopausal group, the toenail iron measure was 8.6% (95% CI : − 14.0, − 3.3) lower per year (Table S2). Women who were postmenopausal at study entry had estimated increases (95% CI) in serum iron (2.3% (0.8, 3.7)) and transferrin saturation (1.6% (0.1, 3.2)) per year increase in age and no evidence of differences in toenail levels for that group either over time (Table 4) or relative to the serum levels (Table S2). We did not find evidence of any trend in iron levels for the women who were still premenopausal at follow-up.

**Table 4:** Regression coefficients for mixed effects models by natural log-transformed iron outcome and menopause status (baseline/follow-up).

Iron status	Menopause status	Age (years)
Serum iron	Post-post	2.25 (0.82, 3.68)
	Pre-post	− 1.44 (− 2.90, 0.01)
	Pre-pre	− 0.21 (− 1.96, 1.55)
Ferritin	Post-post	1.08 (− 2.88, 5.05)
	Pre-post	7.07 (3.65, 10.49)
	Pre-pre	0.51 (− 2.94, 3.96)
Transferrin saturation	Post-post	1.64 (0.09, 3.19)
	Pre-post	− 1.05 (− 2.56, 0.47)
	Pre-pre	0.29 (− 1.59, 2.17)
Nail iron	Post-post	0.63 (− 2.87, 4.13)
	Pre-post	− 3.48 (− 6.12, − 0.84)
	Pre-pre	− 0.20 (− 2.82, 2.42)

Coefficients multiplied by 100.

### Seasonal Patterns in toenail levels

After removing any seasonal variation present in toenail levels, we did not find any substantive changes in the correlations between toenail and serum iron levels with the exception that there were stronger correlations between toenail iron and serum transferrin and serum iron at follow-up. The corrected Spearman correlations at baseline (follow-up) for toenail iron were 0.09 (0.16) for serum iron, − 0.10 (− 0.11) for serum ferritin, and 0.10 (0.20) for serum transferrin saturation. The Spearman correlation (95% CI) between the baseline and follow-up levels for toenail iron was 0.53 (0.36, 0.70), slightly higher than the value of (0.49, (0.31, 0.67)) without correction for season.

## Discussion

Our data do not support an association between serum and toenail iron levels, either at baseline or at follow-up eight years later. Correlations between toenail iron and serum values ranged between − 0.17 for ferritin to 0.09 for serum iron. For the subsample with both baseline and follow-up levels, toenail iron and serum ferritin levels were each moderately repeatable, with rank-based correlation coefficients between baseline and follow-up levels up to about 0.50. Accounting for seasonal variation did not substantively change our results, with the exception of slightly stronger correlations between toenail and serum iron and serum transferrin at follow-up.

Our median results for the serum iron values (Table 1) were comparable to iron status indicators found in representative samples of U.S. women collected as part of the 1999–2002^47^ and 2003–2006 National Health and Nutrition Examination Survey^48^. For example, for U.S. women, the median levels were 53.0 mcg/mL from ages 40–59 years, 1999–2002, and 45.9 mcg/mL for women ages 40–49 years, 2003–2006, for serum ferritin compared to 40.5 mcg/mL at baseline in our sample (Table 1) with a median age at baseline of 45 years (IQR: 42–48), 2003–2009. In women ages 40–59, 1999–2002, serum iron was 78.0 mcg/mL and transferrin saturation was 21.5 percent, compared to the values of 89.5 mcg/mL and 26.0% in our sample at baseline. Toenail iron values in a sample of men and women from the Southeastern U.S., 2004–2012, had a median of 9.0 mcg/g in their control group compared to the 11.8 mcg/g at baseline in our sample. Overall, iron status in this study was comparable to results from U.S. populations.

When estimating change in iron over years of age and considering groups by menopause status change over time, the group of women who had stopped menstruating at study entry showed increases in serum iron and transferrin saturation over time. Women who stopped menstruating between study entry and follow-up demonstrated decreasing serum iron and increasing serum ferritin with increasing time. The increases for ferritin are in line with what would be expected, given the cessation of monthly blood loss caused by menopause^43^. By contrast, we did not find strong evidence that toenail iron changed over age-time for any of the three menopausal groups.

Prior evidence comparing nail and serum iron levels is limited, but comparisons have been made for other trace elements. Selenium findings were mixed, with one study indicating a strong correlation (r = 0.89) between serum and toenail levels^30^ and another study indicating a weak negative correlation (r = − 0.16)^49^. The only other study comparing trace metal levels between serum and toenails was for zinc^50^, and the correlation was not reported, but not statistically significant at an alpha level of 0.05. A small study comparing serum and fingernail iron used a sample of 17 individuals^51^ and reported no correlation between them, but did not provide any statistical analysis and the x-ray fluorescence used to determine toenail iron are not comparable to the mass spectrometry methods used for the toenail samples in this study.

Our own findings do not support the use of toenails as a proxy for serum iron levels. Nevertheless, it is possible that toenails provide a different, but valid, indicator of long-term iron status. One plausible contributor to the poor association between the two types of levels could be that they integrate over different time windows of exposure. The growth of toenails from base to tip can require between 8 and 14 months^52,53^. Clippings are from the end of the toenail, and thus a toenail clipping could reflect a time frame of up to 14 months prior to the collection. This time frame would not be equivalent to that of the serum levels, which tend to reflect more recent iron levels up to the time of the blood draw. However, despite their evident failure to correlate with each other, serum ferritin and toenail levels did both demonstrate better stability across 8 years than did the other serum levels.

The mechanisms by which iron is incorporated into the toenail matrix and into serum involve different biological processes, and that could be another reason for the discrepancy between nail and serum iron levels. For example, iron that is absorbed into the collected portion of nails comes from epidermal cells in the nail bed that differentiate into keratin^54^, which is a different biological process than for serum levels such as ferritin and transferrin saturation. The mechanisms governing the uptake of iron into skin are not well understood^55^, and by extension, the incorporation of iron into the keratinized nail plate is not well understood nor are there any published studies on this topic. These differing uptake mechanisms would be relevant when trying to understand the differences in iron between serum and toenail levels.

For women completing the transition through menopause, iron stores can increase given that they no longer have regular blood loss, which in premenopausal women contributes to lower iron stores and iron deficiency anemia^44^. In this study, ferritin, considered a marker of iron stores given no underlying health conditions^11,13,56^, did increase over time for women who were premenopausal at study entry but had stopped menstruating at the second time point and also for women who were postmenopausal at both time points. In contrast, the toenail iron measure did not show this expected increase.

Factors biasing the toenail sample collection and levels may also contribute to the differences between the serum and toenail levels over time and menopause group change. Aging, related to slower nail growth^57^ and increased nail brittleness^58^, may lead to less toenail mass available for collection and bias measured levels of trace elements^26^. In addition to age, iron itself is an essential element and can influence nail growth^55^. Iron deficiency anemia in the United States is 6% for women who are 50–59 years^59^, and anemia is also associated with brittle nails^60^. Brittleness with increasing age may lead to an underestimation of iron levels in the body.

Also, as mentioned before, the exposure time window represented by iron toenail levels is different from those for serum levels, and, if menopause was recent, then the toenail iron levels could be more indicative of the premenopausal period. The serum levels, considered to reflect a time window much closer to the time of specimen collection, would more represent the later, postmenopausal period, contributing to a mismatch between the two levels. That time difference, however, represents a small fraction of the 8-year interval between collections.

## Strengths and limitations

Strengths of this study include the availability of a subsample with repeated levels over time, which has been lacking in the small number of studies examining associations between nail and serum iron levels. With these repeated levels we also were able to subset the sample into combinations of pre- and postmenopausal groups, allowing for a more complete picture of iron change based on menstrual status, which influences iron levels^42^. We also had a set of serum iron levels not found in other studies, including iron, ferritin, and transferrin saturation, which represent different mechanisms of availability and storage, in our comparisons with toenail iron. We were also able to conduct a sensitivity analysis and assess correlations following removal of seasonal variation in toenail iron values.

In addition to the strengths of the study, we note some limitations. First, we only collected one sample per person at each of the measurement times. Certain levels have more variability than others, implying that repeated samples would provide a more accurate measure. For example, day-to-day variability is evidently higher for serum iron and transferrin saturation than for ferritin in elderly women^17^. Findings for within-person CV also reflect those differences between ferritin and iron^19^. Second, our sample was less than 200 participants, and a larger sample size for this type of study may provide estimates with higher precision. Third, another limitation may be the possibility that this study of women with a first-degree family history of breast cancer who were at least 35 years old at baseline, may not generalize to other populations such as men or different age ranges for women. However, we did find that our between-person coefficients of variation for serum ferritin levels without a natural log-transform (1.03 at baseline and 1.11 at follow-up) were similar to the CV of 0.98 that was reported for the general United States population^18^.

In conclusion, we found little evidence of an association between serum and toenail iron when examining correlations on a cross-sectional basis in a sample of women ages 35–59 at study enrollment. When comparing changes over time, we found the expected increase in ferritin for women whose menstrual cycles stopped during the interval, but no corresponding increase in the toenail levels. The lack of correlations for cross-sectional levels and among their changes across time could be related to different physiologic regulation of these types of levels or temporality of iron storage. These biologic matrices reflect different mechanisms of storage and time windows of exposure, which may be relevant when trying to understand the differences in iron between serum and toenail levels. Although we did not find evidence to support toenails as a proxy that could replace the more invasive serum iron levels, further research as to their properties and what aspects of iron storage they represent are warranted.

### Supplementary Information

Supplementary Information.

## Data Availability

The datasets used and/or analysed during the current study available from the corresponding author on reasonable request (https://sisterstudy.niehs.nih.gov/).
