# Supplementary material for: Toenail and serum levels as biomarkers of iron status in pre- and postmenopausal women: correlations and stability over eight-year follow-up
Source: Sci Rep. 2024 Jan 19;14:1682. doi: 10.1038/s41598-023-50506-5 (PMC10798942; doi:10.1038/s41598-023-50506-5)
Supplement: Supplementary file 1 — Supplementary Information. [file 41598_2023_50506_MOESM1_ESM.pdf]

Supplement

Supplemental text regarding mixed effects models

The following model estimates iron levels from one serum iron outcome and the nail iron outcome, each measured twice over time. We fit the following model separately for each of the three serum outcomes: serum iron, serum ferritin, and serum transferrin saturation.

The mixed effects model includes the natural log transformed serum and nail levels as outcomes and age-time as a covariate with a continuous autocorrelation structure for correlation of residuals over time.

- Model:

$$y_{ijk} = \beta_{0i} + \beta_1 \cdot x_{1ijk} + \beta_{2i} \cdot x_{2ijk} + \beta_3 \cdot x_{1ijk} \cdot x_{2ijk} + \epsilon_{ijk}$$

  - i, j, k are the ith person, jth nail/serum measure and kth time (years)
  - y_{ijk} = natural log transformed iron measure for person i, nail/serum j at time k.
  - β_{0i} = random intercept for person i
    * $\beta_{0i} = \beta_0 + \gamma_{0i}$
  - β_1 = fixed coefficient for age (years)
  - x_{1ijk} = age (years) at measure for person i and nail measure j and time k.
  - β_{2i} = random coefficient for nail for person i
    * $\beta_{2i} = \beta_2 + \gamma_{2i}$
  - x_{2ijk} = binary indicator for nail (1=nail, 0=serum) for person i and nail measure j and time k.
  - ϵ_{ijk} = random error term for person i, nail value j, time k, $\epsilon_{ijk} \sim N(0, \Sigma_{AR})$
  - Σ_{AR} is the continuous autoregressive correlation structure for the residuals, $\sigma^2 \rho^{t_1 - t_2}$, where t_1 and t_2 are age at observation times (age scale) for time 1 and time 2.
  - $(\gamma_{0i}, \gamma_{2i}) \sim N(0, \Sigma_{random})$
    * $\Sigma_{random} = \begin{pmatrix} \tau_0 & \tau_{01} \\ \tau_{01} & \tau_1 \end{pmatrix}$

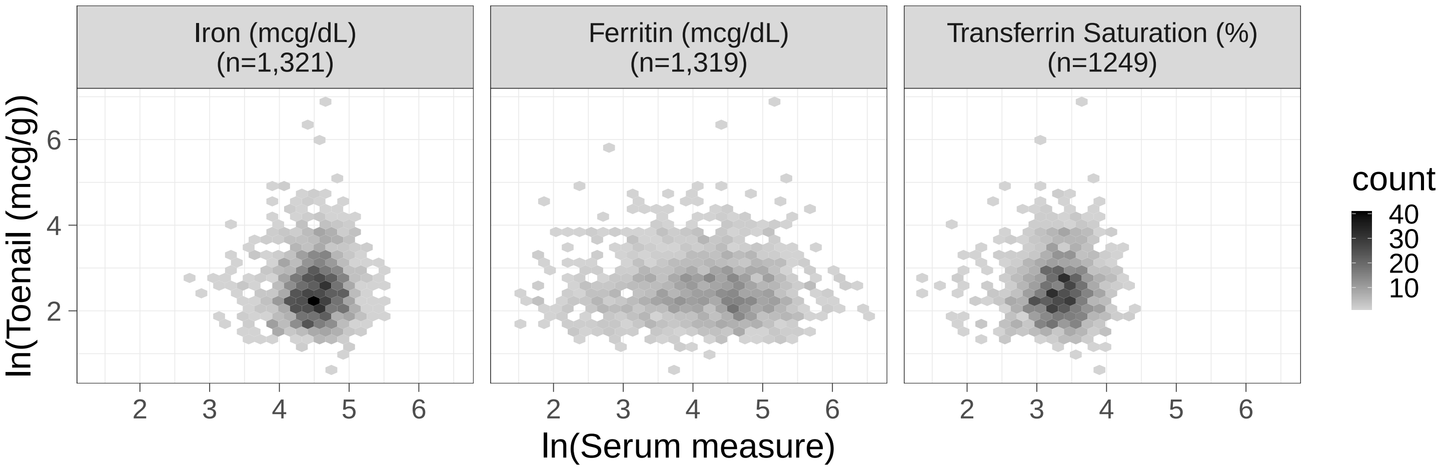

Figure S1: Replication sample: Baseline natural log transformed iron serum versus toenail levels.

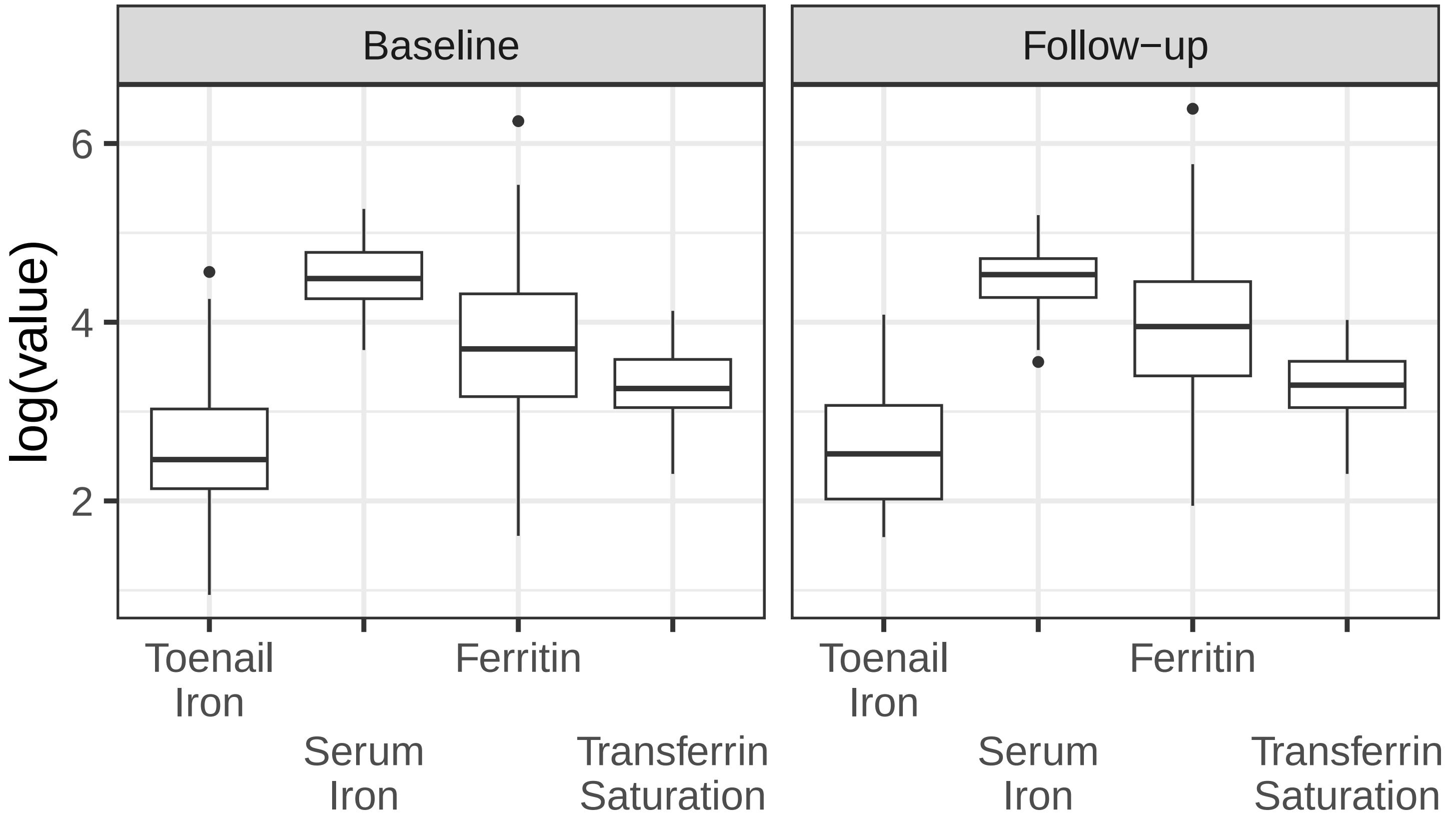

Figure S2: Boxplots of natural log transformed iron serum and nail levels at baseline and follow-up.

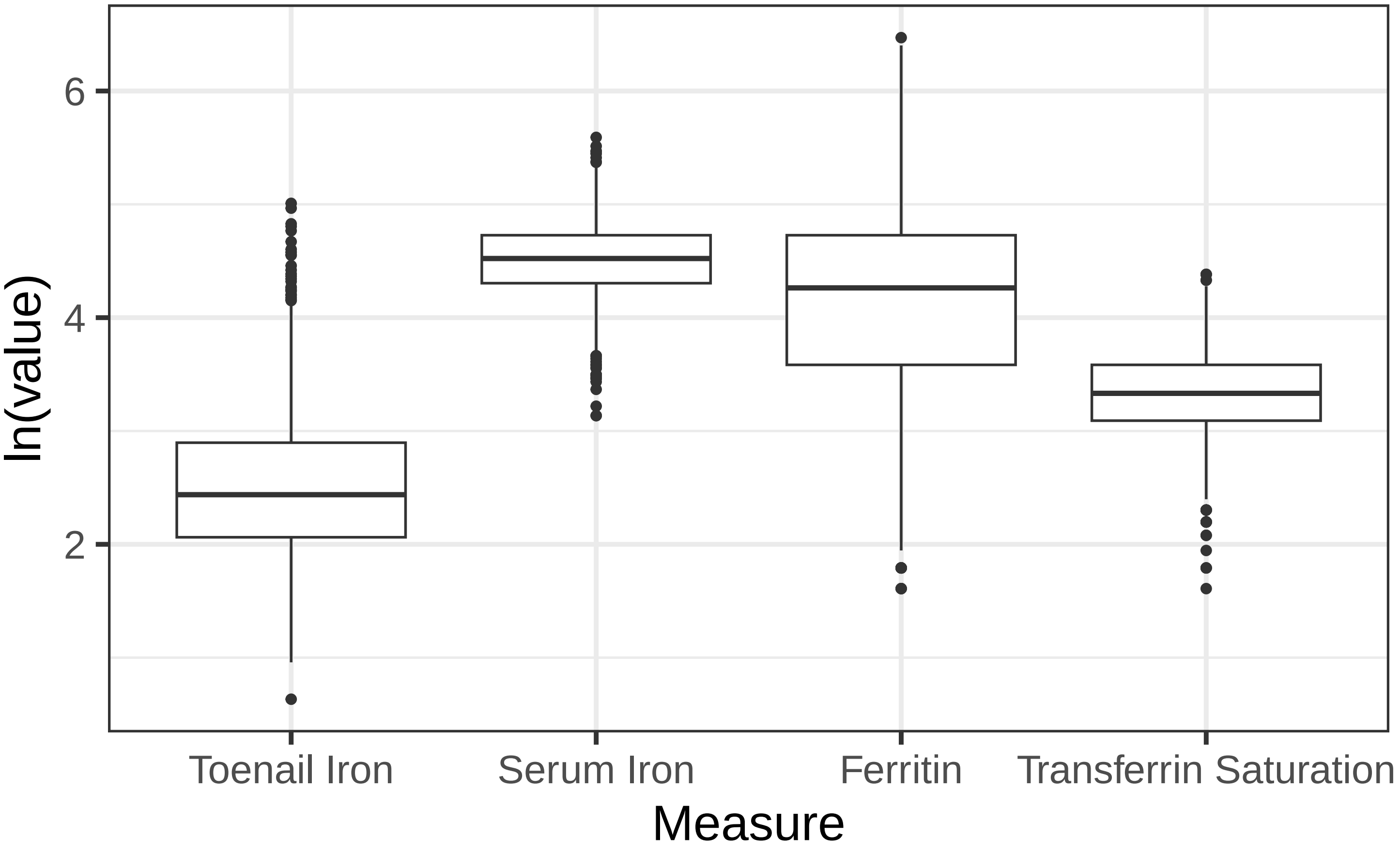

Figure S3: Replication sample: Boxplots of natural log transformed baseline iron serum and nail levels.

Table S1: Replication sample: Spearmean correlations between nails and serum iron values[a] and coefficient of variation for natural log-transformed levels at baseline and follow-up.

Measure	Coefficient of variation	Spearman correlation coefficient
Toenail Iron	0.27	
Serum Iron	0.08	0.04
Ferritin	0.21	-0.01
Transferrin Saturation	0.12	0.04

[a] For natural log-transformed values.

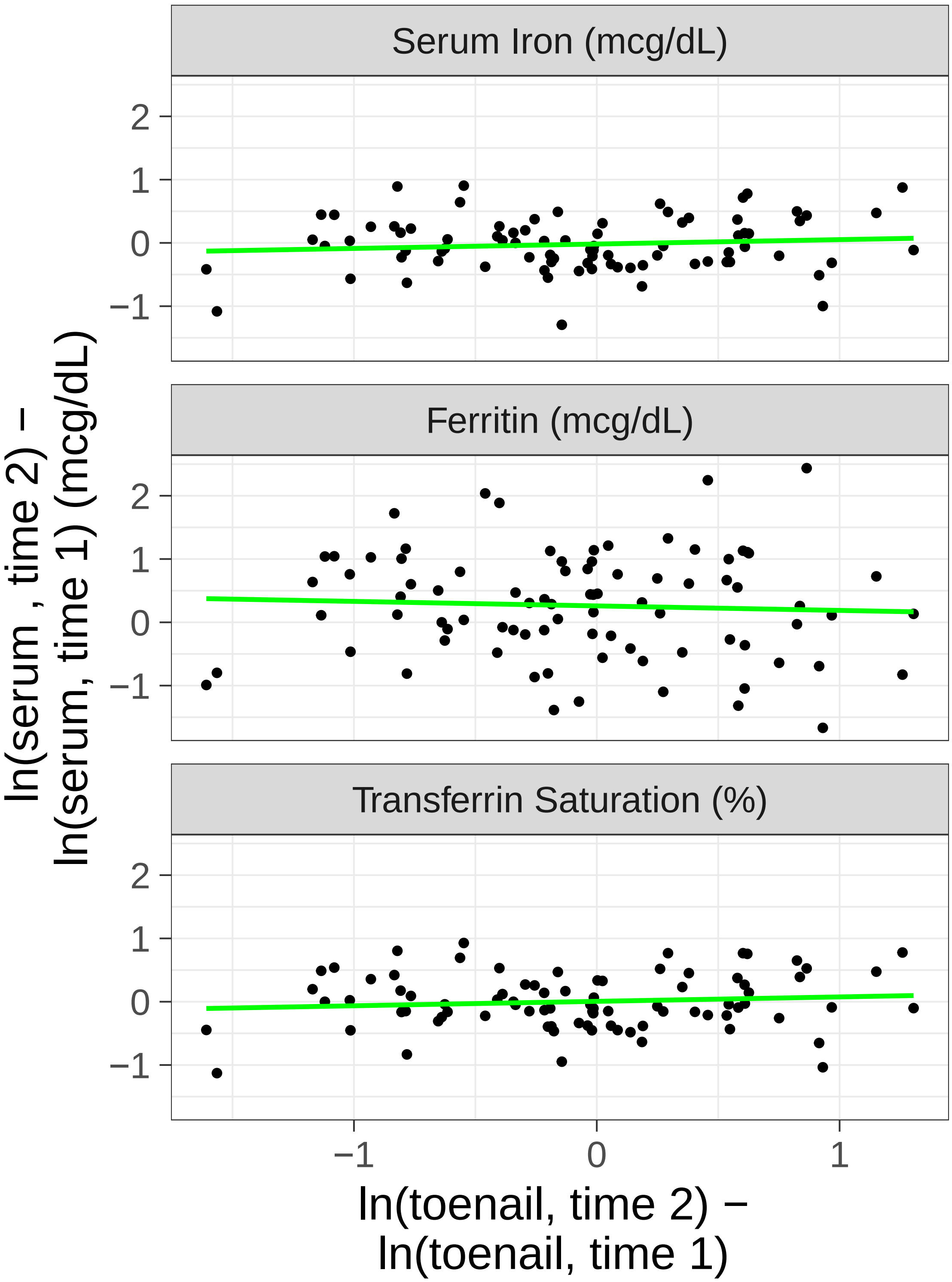

Figure S4: Differences in natural log transformed values between entry and follow-up by nail and serum status. Correlation between the nail iron and serum levels are 0.02 for serum iron, -0.05 for serum ferritin, and 0.02 for transferrin saturation. The solid green line represents the slope from a simple linear regression.

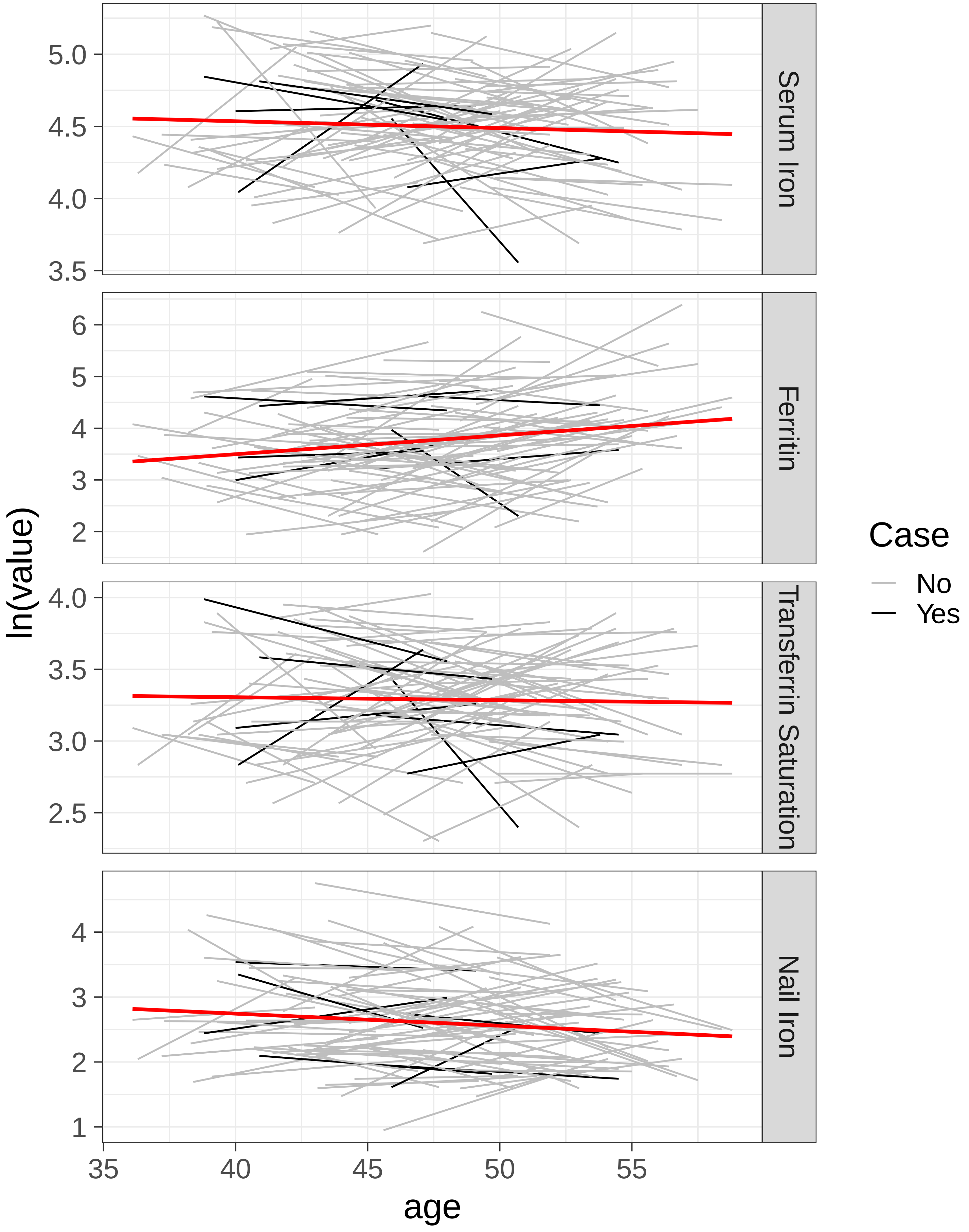

Figure S5: Replication sample: Nail and serum values over time by type of iron measure, menopause status combinations at baseline/follow-up, and case status. The solid red line indicates the fitted line from the mixed effects regression model, and each solid black line represents an individual.

Table S2: Regression coefficients for mixed effects models by iron outcome and menopause status (status at baseline/follow-up)

Serum value	Menopause Status	Intercept	Age	Nail (yes=1 vs. no=0)	Age x Nail
Iron	post/post	3.371 (2.658, 4.083)	0.023 (0.008, 0.037)	-0.860 (-3.188, 1.468)	-0.024 (-0.071, 0.023)
	pre/post	5.204 (4.431, 5.977)	-0.015 (-0.031, 0.000)	-1.050 (-2.954, 0.853)	-0.016 (-0.055, 0.022)
	pre/pre	4.576 (3.786, 5.367)	-0.001 (-0.018, 0.016)	-1.527 (-3.032, -0.021)	-0.007 (-0.040, 0.026)
Ferritin	post/post	3.726 (1.836, 5.616)	0.010 (-0.028, 0.048)	-1.583 (-4.248, 1.083)	-0.004 (-0.057, 0.049)
	pre/post	0.974 (-1.147, 3.095)	0.055 (0.013, 0.098)	3.154 (0.496, 5.812)	-0.086 (-0.140, -0.033)
	pre/pre	3.479 (2.047, 4.911)	0.003 (-0.028, 0.034)	-0.681 (-2.693, 1.330)	-0.005 (-0.049, 0.038)
Transferrin Saturation	post/post	2.485 (1.713, 3.256)	0.017 (0.001, 0.032)	-0.027 (-2.354, 2.299)	-0.017 (-0.064, 0.030)
	pre/post	3.843 (3.026, 4.661)	-0.012 (-0.029, 0.004)	0.257 (-1.687, 2.202)	-0.018 (-0.057, 0.021)
	pre/pre	2.983 (2.021, 3.945)	0.007 (-0.014, 0.028)	-0.160 (-1.549, 1.229)	-0.010 (-0.041, 0.020)
